# Thyroid Fine-Needle Aspiration Biopsy and Thyroid Cancer Diagnosis: A Nationwide Population-Based Study

**DOI:** 10.1371/journal.pone.0127354

**Published:** 2015-05-28

**Authors:** Li-Ying Huang, Ya-Ling Lee, Pesus Chou, Wei-Yih Chiu, Dachen Chu

**Affiliations:** 1 Department of Community Medicine, Taipei City Hospital, Taipei, Taiwan; 2 Institute of Public Health and Community Medicine Research Center, National Yang-Ming University, Taipei, Taiwan; 3 Department of Health Care Management, National Taipei University of Nursing and Health Sciences, Taipei, Taiwan; 4 Department of Neurosurgery, Taipei City Hospital, Taipei, Taiwan; 5 Department of Dentistry, Taipei City Hospital, Taipei, Taiwan; 6 Department of Dentistry, School of Dentistry, National Yang-Ming University, Taipei, Taiwan; 7 Division of Endocrinology and Metabolism, Department of Internal Medicine, National Taiwan University Hospital and National Taiwan University College of Medicine, Taipei, Taiwan; University of Toronto, CANADA

## Abstract

**Background:**

Thyroid cancer is the most common endocrine gland malignancy and fine-needle aspiration biopsy is widely used for thyroid nodule evaluation. Repeated aspiration biopsies are needed due to plausible false-negative results. This study aimed to investigate the overall relationship between aspiration biopsy and thyroid cancer diagnosis, and to explore factors related to shorter diagnostic time.

**Methods:**

This nationwide retrospective cohort study retrieved data from the Longitudinal Health Insurance Database in Taiwan. Subjects without known thyroid malignancies and who received the first thyroid aspiration biopsy after 2004 were followed-up from 2004 to 2009 (n = 7700). Chi-square test, Kaplan-Meier survival analysis, and Cox proportional hazards model were used for data analysis.

**Results:**

Of 7700 newly-aspirated patients, 276 eventually developed thyroid cancer (malignancy rate 3.6%). Among the 276 patients with thyroid cancer, 61.6% underwent only one aspiration biopsy and 81.2% were found within the first year after the initial aspiration. Cox proportional hazards model revealed that aspiration frequency (HR 1.07, 95% CI 1.06–1.08), ultrasound frequency (HR 1.02, 95% CI 1.01–1.03), older age, male sex, and aspiration biopsies arranged by surgery, endocrinology or otolaryngology subspecialties were all associated with shorter time to thyroid cancer diagnosis.

**Conclusions:**

About 17.4% of thyroid cancer cases received more than two aspiration biopsies and 18.8% were diagnosed one year after the first biopsy. Regular follow-up with repeated aspiration or ultrasound may be required for patients with clinically significant thyroid nodules.

## Introduction

Thyroid cancer is the most common endocrine system malignancy and accounts for 1–2% of all human cancers [1–4]. In the United States, the age-adjusted incidence rate for thyroid cancer is 12.9/100,000 per year [5], which is approximately 8.49/100,000 in Taiwan [6]. Recent studies have disclosed a worldwide trend of increasing thyroid cancer incidences [1, 7, 8], perhaps due to improvements in small tumor detection [8].

Most differentiated thyroid cancers harbor a rather favorable prognosis if identified in the early stage [9]. Without distant metastases upon initial diagnosis, the mortality rate may be as low as 6% and 10% in a 30-year follow-up for papillary thyroid cancer and follicular thyroid cancer, respectively [10]. However, the 30-year mortality rate may go up to 65% among patients with advanced stage IV cancer [10]. The risk of recurrence and death also significantly increases with advanced age, extra-thyroid invasion, or distant metastases on initial diagnosis [4, 9, 11, 12]. Hence, earlier diagnosis is essential.

In international guidelines, high-resolution ultrasonography and fine-needle aspiration biopsy are recommended as first-line evaluation tools of thyroid nodules [13, 14]. Although thyroid ultrasonography is convenient and non-invasive, the ultrasound features are not adequately sensitive to detect all thyroid cancers [14]. Hence, thyroid aspiration biopsies are the most important diagnostic tool for thyroid malignancies.

The accuracy rate of thyroid fine-needle aspiration biopsy has been extensively explored [15–18], with mean sensitivity and specificity of approximately 83% and 92%, respectively [19]. The reported false-negative rate, however, ranges from 1% to 21% [19, 20] and the adequacy of samples may be technician-dependent [21]. Accordingly, discrepancies among the initial and subsequent aspiration results are not uncommon [13, 20]. False-negative thyroid aspirations can delay thyroid cancer treatment and may adversely affect outcomes [20]. Thus, repeated aspiration during follow-up is often necessary.

Nonetheless, there is paucity of data regarding the clinical details of aspiration biopsy. Moreover, large-scale studies with sufficient patient number and long-term follow-ups are scarce. Hence, using the nationwide longitudinal health insurance database in Taiwan, this study aimed to (1) explore the overall malignancy rate among patients who underwent thyroid aspiration biopsy; (2) demonstrate the frequency of aspirations and the time from the first aspiration to thyroid cancer diagnosis; and (3) explore factors affecting the time from the first aspiration to thyroid cancer diagnosis.

## Material and Methods

### Data Source

Data was retrieved from Taiwan’s Longitudinal Health Insurance Database (LHID 2005) covering the period of January 1, 2002 to December 31, 2009. The LHID 2005 was formed through the cooperation of the National Health Insurance (NHI) Administration and the National Health Research Institute (NHRI) of Taiwan. It contained original in-patient and out-patient claims data of 1,000,000 randomly sampled beneficiaries of the NHI program in the year 2005.

The NHI is a compulsory insurance program that centralizes expenses on healthcare funds. Began in 1995, the program has now enrolled up to 99% of Taiwan inhabitants. The LHID research database contains patients’ medical orders, operative procedures, and clinical diagnoses, with diagnostic codes based on the International Classification of Diseases, Ninth Revision, Clinical Modification Code (ICD9-CM). Age and sex distribution in this sampled sub-population resembles that of the entire population of NHI. All of the patients’ identifications have been encrypted to safeguard privacy and access to data analysis has been approved by the NHRI.

The Taipei City Hospital Institutional Review Board approved the study (No. TCHIRB-1020820-E) and waived the need for written informed consent.

### Study Samples

**Fig 1:**
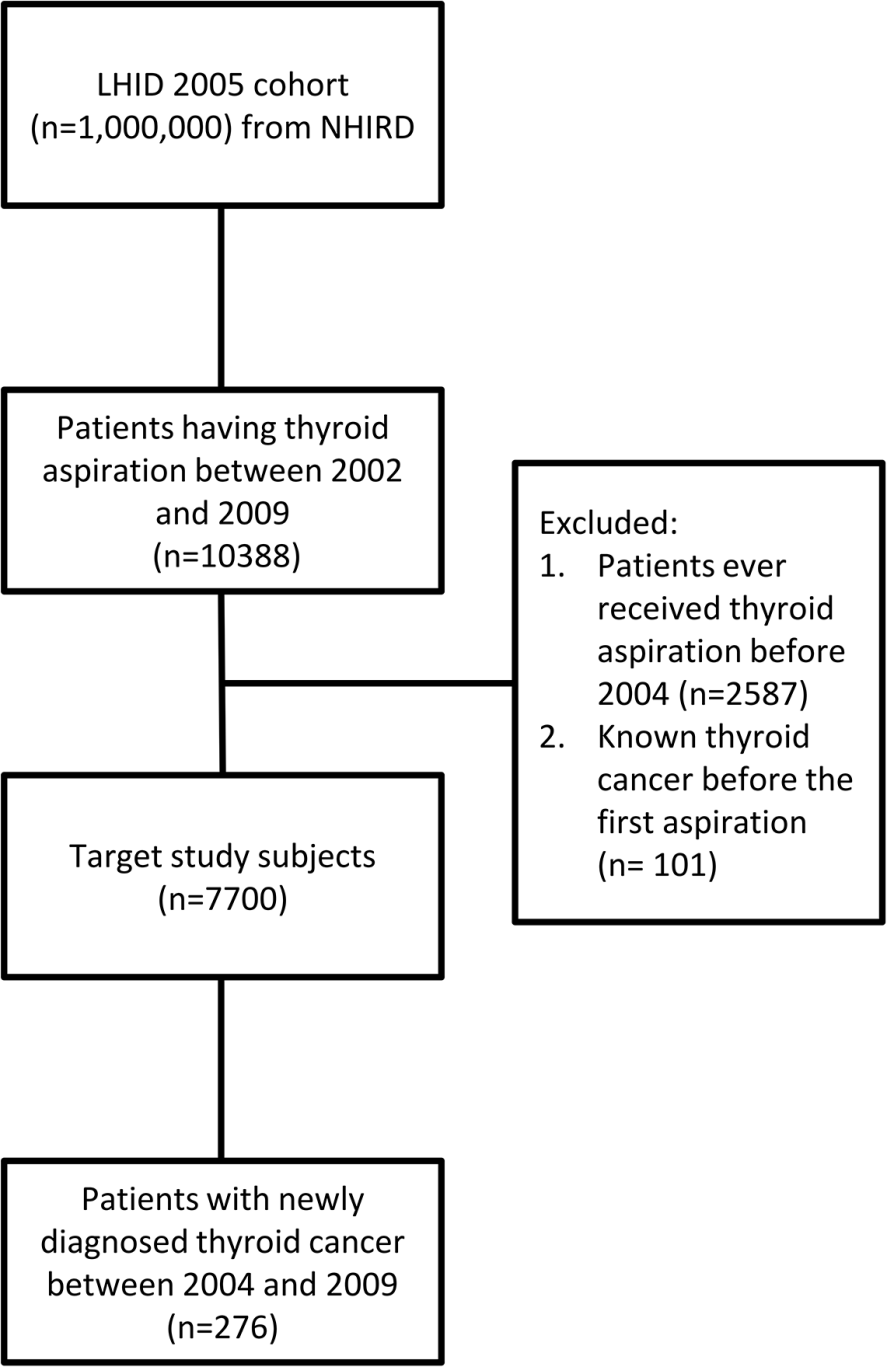
Selection of Study Patients.

This nationwide, population-based, retrospective cohort study followed the subjects who underwent thyroid aspiration biopsies between 2002 and 2009 (n = 10388). Patients with previous thyroid aspiration before 2004 (n = 2587) and known thyroid cancer (n = 101) before their first biopsy were excluded, resulting in a final cohort of 7700 subjects (Fig 1).

The initial diagnosis was defined as the ICD9-CM code used on the date when the first thyroid aspiration study was arranged, including thyrotoxicosis (242, 242.4X, 242.8X, 242.9X); hypothyroidism (243, 244.X); diffuse goiter (242.0X); nodular goiter (241, 241.0, 241.9, 242.1X, 242.3X); multinodular goiter (241.1, 242.2X); unspecified goiter (240.X, 246.1); neoplasm of thyroid (226); thyroid cyst (246.2); acute thyroiditis (245.0); subacute thyroiditis (245.1); chronic lymphocytic thyroiditis (245.2); other thyroiditis (245, 245.3, 245.4, 245.8, 245.9); other thyroid disorders (017.5X, 122.2, 246, 246.0, 246.3, 246.8, 246.9, 790.94, 794.5, 848.2, 874.2, 874.3).

During follow up, there were 276 thyroid cancer patients confirmed by a major illness registration of thyroid cancer (ICD9-CM code 193) in the NHRI database. The index dates for thyroid cancer diagnoses were identified as the date of their first clinical record of thyroid cancer or the date when the patients were issued a major illness registration due to thyroid cancer, whichever occur first.

Ultrasound frequency was defined as thyroid ultrasound arranged after the first aspiration and without concomitant aspiration, before being diagnosed as thyroid cancer. The mean aspiration interval (MAI) was used to evaluate follow up intensity and was calculated by dividing the overall follow-up period by the overall aspiration numbers. Subjects were divided into four categories according to their follow-up intensity: (1) low intensity, MAI >2 years; (2) medium intensity, MAI 1–2 years; (3) high intensity, MAI 0.5–1 year; and (4) extremely high intensity, MAI <0.5 year.

### Statistical Analysis

This study used the SAS statistical package, version 9.2 (SAS Institute, Cary, NC) and JMP 5.0 to perform all statistical analysis. Chi-square test was used for between group comparisons. Malignancy rate and time and aspiration number before thyroid cancer diagnoses were displayed as percentage and cumulative percentage, respectively. Median time to thyroid cancer diagnosis was calculated using the Kaplan-Meier survival analysis and log-rank test. A Cox proportional hazards model was used to determine predictors of time needed for cancer diagnosis. A hazard ratio (HR) >1.0 indicated an association with shorter time to thyroid cancer diagnosis, whereas an HR <1.0 indicated an association with longer time to thyroid cancer diagnosis. And a *p*-value < 0.05 is considered statistically significant.

## Results

Among the 7700 aspirated patients, 69.0% were aged 30–59 years. The leading initial ICD-9-CM diagnosis was unspecified goiter (31.9%), followed by nodular goiter (24.4%) and multi-nodular goiter (7.0%) (Table 1). There were 276 patients who developed thyroid cancer (malignancy rate 3.6%) during the 6-year follow-up. In the subgroup of patients initially diagnosed as thyroid goiter, cystic, or neoplastic lesion (n = 5689), the malignancy rate was slightly higher at 4.0%. Approximately two malignant cases were identified for every one hundred thyroid aspirations performed (2.0%).

**Table 1:** Baseline characteristics of 7700 patients who underwent thyroid fine-needle aspiration biopsy.

		Total	Benign	Malignancy	*p*-value
		(n = 7700)	(n = 7424)	(n = 276)	
Age					0.003
	< 20	168 (2.2)	162 (96.4)	6 (3.6)	
	20~29	671 (8.7)	641 (95.5)	30 (4.5)	
	30~39	1302 (16.9)	1240 (95.2)	62 (4.8)	
	40~49	1945 (25.3)	1863 (95.8)	82 (4.2)	
	50~59	2061 (26.8)	1998 (96.9)	63 (3.1)	
	60~69	978 (12.7)	954 (97.5)	24 (2.5)	
	70~79	481 (6.2)	472 (98.1)	9 (1.9)	
	> = 80	94 (1.2)	94 (100.0)	0 (0.0)	
Gender					0.43
	Male	1291 (16.8)	1240 (96.0)	51 (4.0)	
	Female	6409 (83.2)	6184 (96.5)	225 (3.5)	
Initial Diagnosis					0.004
	Thyrotoxicosis	451 (5.9)	445 (98.7)	6 (1.3)	
	Hypothyroidism	95 (1.2)	94 (98.9)	1 (1.1)	
	Diffuse goiter	304 (3.9)	300 (98.7)	4 (1.3)	
	Nodular goiter	1878 (24.4)	1797 (95.7)	81 (4.3)	
	Multinodular goiter	537 (7.0)	513 (95.5)	24 (4.5)	
	Unspecified goiter	2459 (31.9)	2365 (96.2)	94 (3.8)	
	Neoplasm of thyroid	379 (4.9)	360 (95.0)	19 (5.0)	
	Thyroid cyst	132 (1.7)	129 (97.7)	3 (2.3)	
	Acute thyroiditis	4 (0.1)	3 (75.0)	1 (25.0)	
	Subacute thyroiditis	80 (1.0)	80 (100.0)	0 (0.0)	
	Chronic lymphocytic thyroiditis	127 (1.6)	125 (98.4)	2 (1.6)	
	Other thyroiditis	31 (0.4)	30 (96.8)	1 (3.2)	
	Other thyroid disorders	588 (7.6)	567 (96.4)	21 (3.6)	
	Not specified*	635 (8.2)	616 (97.0)	19 (3.0)	
Hospital					0.69
	Medical Center	3658 (47.5)	3523 (96.3)	135 (3.7)	
	Regional Hospital	2757 (35.8)	2658 (96.4)	99 (3.6)	
	Area Hospital	1128 (14.6)	1089 (96.5)	39 (3.5)	
	Clinic	157 (2.0)	154 (98.1)	3 (1.9)	
Physician					0.006
	Endocrinology	4756 (61.8)	4607 (96.9)	149 (3.1)	
	Surgery	1044 (13.6)	988 (94.6)	56 (5.4)	
	Internal medicine	909 (11.8)	883 (97.1)	26 (2.9)	
	Otolaryngology	650 (8.4)	620 (95.4)	30 (4.6)	
	Family medicine	288 (3.7)	276 (95.8)	12 (4.2)	
	Others	53 (0.7)	50 (94.3)	3 (5.7)	

* Thyroid related diagnoses were not recorded.

Among the 276 thyroid cancer patients who underwent thyroid aspiration evaluation, 70.7% of the thyroid malignancies were identified within half a year. The cumulative diagnosis rate increased to 81.2% and 92.0% by the end of year one and year two, respectively. There were 8.0% and 3.6% of thyroid cancer cases undiagnosed at the end of year two and year three, respectively (Table 2).

**Table 2:** Time from first thyroid fine-needle aspiration to thyroid cancer diagnosis.

Time to diagnosis	No. of patients (n)	Cancer (%)	Cumulative (%)	Undiagnosed (%)
< 1 month	65	23.6	23.6	76.4
1~3 month	100	36.2	59.8	40.2
3~6 month	30	10.9	70.7	29.3
0.5~1 year	29	10.5	81.2	18.8
1~2 year	30	10.9	92.0	8.0
2~3 year	12	4.3	96.4	3.6
3~4 year	6	2.2	98.6	1.4
4~5 year	1	0.4	98.9	1.1
5~6 year	3	1.1	100.0	0.0

Among the 276 malignant cases, 61.6% underwent only one aspiration biopsy, while 21.0% received a second aspiration before their final diagnoses of cancer and 9.8% of patients need more than three aspirations before cancer was diagnosed (Table 3).

**Table 3:** Number of thyroid fine-needle aspiration before thyroid cancer diagnosis.

Aspiration(s)	No. of patients (n)	Cancer (%)	Cumulative (%)	Undiagnosed (%)
1	170	61.6	61.6	38.4
2	58	21.0	82.6	17.4
3	21	7.6	90.2	9.8
4	16	5.8	96.0	4.0
5	6	2.2	98.2	1.8
6	3	1.1	99.3	0.7
> 6	2	0.7	100.0	0.0

The median time to cancer diagnosis, calculated by Kaplan-Meier survival analysis, was shortest among patients with extremely high follow-up intensity (0.13 years), followed by patients with high (1.21 years), medium (1.88 years), and low (3.25 years) follow-up intensity. These were statistically different (*p*<0.0001) (Table 4).

**Table 4:** Median time to thyroid cancer diagnosis among patients with thyroid fine-needle aspirations (n = 276/7700).

Patient group	Mean aspiration interval	Patients	Thyroid Cancer (%)	Median time to diagnosis (yr)*
Low intensity	≧ 2 years	3236	5 (0.2)	3.25 (2.03, 4.30)
Medium intensity	1~2 years	1787	18 (1.0)	1.88 (1.15, 2.47)
High intensity	0.5~1 year	1255	26 (2.1)	1.21 (0.67, 1.85)
Extremely high intensity	< 0.5 year	1422	227 (16.0)	0.13 (0.10, 0.15)
Overall		7700	276 (3.6)	

* Log-Rank Test:*p* < 0.0001

In the Cox proportional hazard model, older age (HR 1.01, 95% CI 1.00–1.02), male sex (HR 1.18, 95% CI 1.00–1.38), higher aspiration frequency (HR 1.07, 95% CI 1.06–1.08), higher ultrasound frequency (HR 1.02, 95% CI 1.01–1.03), and physician specialty of surgery (HR 2.55, 95% CI 1.39–5.02), endocrinology (HR 2.58, 95% CI 1.45–4.95), and otolaryngology (HR 2.47, 95% CI 1.25–5.15) were all associated with shorter time to thyroid cancer diagnosis (Table 5).

**Table 5:** Cox proportional hazard model for time to thyroid cancer diagnosis (n = 276).

	HR*	95% CI
Age	1.01	1.00–1.02
Gender		
Male	1.18	1.00–1.38
Female	1.00	
Initial diagnosis		
Thyrotoxicosis	0.84	0.32–1.86
Hypothyroidism, Hashimoto	0.81	0.19–2.27
Goiter, Neoplasm	1.08	0.78–1.52
Others	1.00	
Aspiration frequency	1.07	1.06–1.08
Ultrasound frequency	1.02	1.01–1.03
Hospital		
Center, Regional	1.20	0.83–1.78
Area, Clinic	1.00	
Physician		
Surgery	2.55	1.39–5.02
Endocrine	2.58	1.45–4.95
ENT	2.47	1.25–5.15
Internal medicine	1.71	0.87–3.52
Others	1.00	

* A hazard ratio (HR) >1.0 indicated an association with shorter time to thyroid cancer diagnosis, whereas an HR <1.0 indicated an association with longer time to thyroid cancer diagnosis.

## Discussion

In this study, there is a malignancy rate of 3.6% among patients who underwent thyroid aspiration biopsy. Among the malignant cases, 61.6% have only one aspiration biopsy prior to diagnosis and 81.2% have been identified within one year after the first aspiration biopsy. The frequency of aspiration and ultrasonography, as well as older age, male sex, and aspiration biopsies arranged by surgery, endocrinology, or otolaryngology subspecialties are associated with a shorter time to thyroid cancer diagnosis.

Many studies have investigated the malignancy rate of patients who underwent thyroid aspiration biopsies. The malignancy rate ranges from 1.6% to 14.9% [14, 22, 23]. Yet thyroid cancer incidence may vary by age, sex, ethnic group, and geographical area [5, 24, 25]. This variability may result from differences in the underlying characteristics of the enrolled subjects, as well as the aggressiveness of screening. The malignancy rate in the present study (3.6%) is similar to that of a northern Taiwan medical center report (3.9%, 858/21748 cases) [26].

The reported sensitivity of thyroid fine needle aspiration may be as low as 65% in some studies [19]. Among the 276 thyroid cancer patients in this series, 38.4% received more than one aspiration before cancer was diagnosed. This may result from the known pitfalls in thyroid aspiration, including inappropriate target selection [13], inadequate sampling [13, 15], gray zones in thyroid cytology interpretation [13, 27], and newly-developed tumor. In a retrospective study, 46.7% of the aspirated thyroid cancers were initially concluded as benign or insufficient for diagnosis [28]. The initially non-diagnostic rates may be as high as 10–20% [29], and the proportion of non-diagnostic specimens increased with greater cystic components of the nodules [30]. When repeated aspiration was performed, up to 38% of the initial non-diagnostic nodules may remain non-diagnostic [29]. Furthermore, the malignancy rates were 11.4% and 11.9% among cases with initial non-diagnostic and indeterminate fine needle aspiration biopsies in one study [31]. In short, a proportion of patients are likely to experience delayed or missed diagnoses if only one aspiration is done. Compared with single FNAB, sequential biopsy was reported to increase the diagnostic sensitivity by 13.8% and specificity by 6.2%, and reduce the false positive/negative results by 14.2% [32]. Hence, repeated aspirations seem necessary to improve the overall diagnostic accuracy. This may be crucial for physicians to consider for possible false-negative results in thyroid aspiration and to highlight the importance of regular follow-up.

There is still no consensus on the optimal timetable for repeat aspiration of a thyroid nodule [13, 14, 33]. In initially benign cases, some suggest routine repeat biopsy during subsequent follow-up [13, 34]. Others recommend repeat aspiration only when there is evidence of nodule growth or suspicious sonographic features [14, 33]. Using Kaplan-Meier survival analysis, a higher aspiration frequency is significantly associated to shorter median time to thyroid cancer diagnosis. Although such observations may partly be the result of patient and physician selection, the finding may provide a better understanding of the approximate time lag from different aspiration intervals. Using Cox regression model in adjusting for confounders, the same results are revealed. These support the more aggressive approach of routine repeated biopsy mentioned in the American Association of Clinical Endocrinologists (AACE), Associazione Medici Endocrinologi (AME), and European Thyroid Association (ETA) guidelines [13]. Nonetheless, the ideal re-aspiration timeframe warrants further investigation.

Older age and male sex are established risk factors for malignant thyroid nodule and worse thyroid cancer prognoses [9, 13, 35]. Male sex is also associated with larger tumor size and more advanced tumor stages [35–37]. These may presumably affect the physician’s management decisions, thereby leading to the earlier diagnoses in men and in the elderly in this study.

In addition, after adjusting for the effects of the frequency of aspiration, an increased frequency of ultrasonography is also an independent prognostic factor for early diagnosis. In other words, high-resolution ultrasonography may provide additional benefits in the diagnosis of thyroid cancer and may serve as a substitute exam if routine repeat biopsy is not desired.

The main strength of this research is its long-term nationwide evaluation of the overall diagnosis of thyroid cancer using aspiration biopsy. The longitudinal health insurance database in Taiwan allows researchers to trace persons over time and across hospitals, grounded on a population-based record. Hence, the results possess superior reliability and generalizability than single-center studies.

However, the current study also has limitations. First, it is unable to specify the exact thyroid cancer subtype. Taiwan’s thyroid cancer distribution (male: 83.89% papillary, 8.23% follicular thyroid cancer; female: 89.99% papillary, 5.82% follicular thyroid cancer [6]) is very similar to that in most countries [2, 38, 39], so the results may be generalized to majority of the clinical settings. Second, information regarding individual ultrasound features, risk factors for thyroid cancer, actual needle size and technique (with or without ultrasound guidance) used during thyroid puncture are either unrecorded or not completely distinguishable from the LHID database. Third, the LHID contains the clinical, but not the cytological diagnoses of patients. Methods for cytological preparation, ancillary studies and cytological classification system adopted (Bethesda or others) are not available as well. However, most aspirated thyroid tissues are directly smeared (for Papanicolaou stain or Liu’s stain) without further split sample comparison or ancillary studies in Taiwan, and smears are reviewed by certified cytopathologists. Therefore, although studies have shown inconsistent diagnostic ability of direct smears and liquid-based cytology [40–43], and that the classification variety may possibly affect results, the effect should be limited and can be minimized by statistic adjustment in this study. Further primary data analysis is warranted for related investigation.

In conclusion, many thyroid cancers can be identified within half a year to one year or after 1–3 aspirations, although nearly 40% of thyroid cancers may remain undiagnosed after the first aspirations. Hence, it is crucial to remember the limitation of the examinations and stress the importance of regular follow-up.
